# Improving product quality and productivity of an antibody-based biotherapeutic using inverted frustoconical shaking bioreactors

**DOI:** 10.3389/fbioe.2024.1352098

**Published:** 2024-03-22

**Authors:** Xuekun Wang, Jin Xu, Qingcheng Guo, Zhenhua Li, Jiawei Cao, Rongrong Fu, Mengjiao Xu, Xiang Zhao, Fugui Wang, Xinmeng Zhang, Taimin Dong, Xu Li, Weizhu Qian, Shen Hou, Lusha Ji, Dapeng Zhang, Huaizu Guo

**Affiliations:** ^1^ State Key Laboratory of Macromolecular Drugs and Large-scale Manufacturing, School of Pharmaceutical Sciences, Liaocheng University, Liaocheng, China; ^2^ NMPA Key Laboratory for Quality Control of Therapeutic Monoclonal Antibodies, Shanghai, China; ^3^ State Key Laboratory of Macromolecular Drugs and Large-scale Manufacturing, School of Pharmaceutical Sciences, Wenzhou Medical University, Wenzhou, China; ^4^ Taizhou Mabtech Pharmaceuticals Co., Ltd., Taizhou, China; ^5^ State Key Laboratory of Macromolecular Drugs and Large-scale Manufacturing, Shanghai Zhangjiang Biotechnology Co., Ltd., Shanghai, China

**Keywords:** inverted frustoconical shaking bioreactor, stirred bioreactor, antibody-based biotherapeutic, scale-up production, productivity

## Abstract

The Chinese hamster ovarian (CHO) cells serve as a common choice in biopharmaceutical production, traditionally cultivated in stirred tank bioreactors (STRs). Nevertheless, the pursuit of improved protein quality and production output for commercial purposes demand exploration into new bioreactor types. In this context, inverted frustoconical shaking bioreactors (IFSB) present unique physical properties distinct from STRs. This study aims to compare the production processes of an antibody-based biotherapeutic in both bioreactor types, to enhance production flexibility. The findings indicate that, when compared to STRs, IFSB demonstrates the capability to produce an antibody-based biotherapeutic with either comparable or enhanced bioprocess performance and product quality. IFSB reduces shear damage to cells, enhances viable cell density (VCD), and improves cell state at a 5-L scale. Consequently, this leads to increased protein expression (3.70 g/L vs 2.56 g/L) and improved protein quality, as evidenced by a reduction in acidic variants from 27.0% to 21.5%. Scaling up the culture utilizing the Froude constant and superficial gas velocity ensures stable operation, effective mixing, and gas transfer. The IFSB maintains a high VCD and cell viability at both 50-L and 500-L scales. Product expression levels range from 3.0 to 3.6 g/L, accompanied by an improved acidic variants attribute of 20.6%–22.7%. The IFSB exhibits superior productivity and product quality, underscoring its potential for incorporation into the manufacturing process for antibody-based biotherapeutics. These results establish the foundation for IFSB to become a viable option in producing antibody-based biotherapeutics for clinical and manufacturing applications.

## Introduction

The biopharmaceutical market, especially for antibody-based biotherapeutics, has experienced rapid growth due to their highly therapeutic effects and excellent targeting abilities. According to a report by Mordor Intelligence, the market was valued at USD 325.17 billion in 2020 and is projected to reach USD 496.71 billion by 2026 (Makurvet, 2021).

Mammalian expression systems serve as platforms to produce biotherapeutics, particularly those based on antibodies. Chinese hamster ovarian (CHO) cells stand out as a popular choice. Other mammalian expression systems encompass rodent cell lines such as NS0, BHK, and Sp2/0, as well as human cell lines like HEK293 and PER. (Wurm, 2004). CHO cells are favored over other cell types due to their stable growth in suspension cultures with chemically defined media, their capacity for post-translational modifications (PTMs) similar to those in humans, and their ease of genetic engineering for the production of high-quality human protein products (Fischer et al., 2015).

The primary types of bioreactors currently employed in the production of antibody-based biotherapeutics for mammalian cell cultures include stirred tank bioreactors (STRs) (Ackermann et al., 2022), bubble column bioreactors (Humbird et al., 2017), air-lift bioreactors (Zhang et al., 2017), and wave mixed bioreactors (Imseng et al., 2014). Among these, STRs dominate the majority of large-scale applications. The critical factor in bioreactors lies in the efficient transfer of oxygen and other nutrients, ensuring a homogeneous distribution of cellular and component concentrations in the medium, and maintaining a consistent culture environment for cell growth, encompassing pH, temperature, and dissolved oxygen (Jia et al., 2008). STRs achieve this through the rotation of agitator paddles, propelling the liquid phase for mixing. Additionally, STRs incorporate mixing systems, such as gas spargers for air, oxygen, and carbon dioxide replenishment, along with baffles for enhanced performance (Marks, 2003). However, the shear generated by the stirring paddles and gas bubbling in STRs can inflict considerable damage on mammalian cells, which are particularly susceptible to shear due to their lack of cell wall support. Consequently, achieving a high level of viable cell density (VCD) in mammalian cells within STRs proves challenging, acting as a bottleneck for their further development (Collins et al., 1998; Li et al., 2010). Therefore, there is a pressing need for innovative bioreactor types to enhance protein quality and production output for commercial purposes, and to provide increased production flexibility, especially for products requiring large-scale production, such as antibody-drug conjugates, or for clinical trials.

Inverted frustoconical shaking bioreactors (IFSBs), featuring a conical bottom that distinguishes them markedly from common orbitally shaken bioreactor (OSR), are not commonly employed in the production of antibody-based biotherapeutics (Hang et al., 2011). They function by circulating liquid along a defined path to achieve mixing without generating a high-intensity shear force. Nanoscale dissolvable microbubbles are generated by repeatedly flushing the inner surface of the culture bag with a medium torrent induced by mechanical oscillation. This ensures constant mixing and complete gas transfer, supplying each cell with adequate oxygen for normal growth and metabolism. IFSB employs a unique gas transfer mechanism that mitigates cell damage associated with traditional bubbling bioreactors. Studies indicate that the shear force produced by oscillating-shaken bioreactors, akin to IFSB, does not surpass the threshold for cell damage (Zhu et al., 2018). Moreover, IFSB operates without sparging, eliminating the risk of bubble-induced cell damage, making it well-suited for mammalian cell culture (Klöckner et al., 2013). The mixing principle of IFSB resembles that of a shake flask. The flow field in the bioreactor is homogeneous and shares a similar geometry with a shake flask, facilitating scalability through the application of the principle of equality of the Froude number (Thompson et al., 2019).

Limited information is available regarding the production of antibody-based biotherapeutics using IFSB with CHO cells, and their impact on process performance and product quality. This study aimed to investigate the productivity and quality of monoclonal antibodies produced through IFSB cultivation with CHO cells. Additionally, we assessed the scalability of IFSB from 5 to 50 and 500 L. The results revealed that the 5-L scale IFSB exhibited higher VCD and productivity, with improved quality in the acidic variants of the antibody-based biotherapy compared to STR. Moreover, as the IFSB was scaled up, the VCD, productivity, and protein quality remained comparable to those at the 5-L scale. These findings suggest that IFSB has the potential to produce antibody-based biotherapeutics with bioprocess performance and product quality that are either comparable to or better than traditional methods. Furthermore, the study demonstrates the scalability of IFSB, reinforcing its viability for large-scale production.

## Materials and methods

### Experimental materials

The experimental materials comprised various instruments and equipment. These included a 5-L STR (SRT-5, Bioengineering AG, Wald, Switzerland), as well as 5-L, 50-L, and 500-L IFSB vessels (IFSB-5, IFSB-50, IFSB-500, Zhejiang JYSS Bio-Engineering Co., Ltd., Zhejiang, China). Other essential apparatus included a carbon dioxide oscillation incubator (Infors HT, Bottmingen, Switzerland), Vi-Cell Viability Analyzer (Beckman Coulter, California, United States), Microplate reader (Bio-Rad Laboratories, California, United States), Autodesk Prime protein purification system (Zhejiang JYSS), Protein A affinity HPLC (GALAK Wuxi, China), IEX-HPLC (Thermo Fisher Scientific, MA, US), and SEC-UPLC (Waters, MA, US). Reagents for cell culture, including the growth medium, were sourced from Shanghai Sinomab Biotechnology Co., Ltd. Additionally, anhydrous glucose was procured from Shandong Xiwang Pharmaceutical Co., Ltd., and sodium bicarbonate from Nanchang Baiyun Pharmaceutical Co., Ltd. The kit for measuring nutrients was obtained from the Nanjing Jiancheng Bioengineering Institute.

### Scale-up strategy

In this study, we initially assessed the process performance parameters and product quality attributes involved in the preparation of antibody-based biotherapy on a 5-L scale using STR-5 and IFSB-5. The capability of IFSB-5 and its associated consumables in the production of the antibody-based biotherapeutic is assessed, providing key technical data for scaling up cell culture. Rather than opting for a direct scale-up from the 5-L scale to the 500-L scale, we chose a more conservative approach. This involved an intermediate step utilizing a 50-L bioreactor, followed by the eventual transition to the 500-L bioreactor. This stepwise approach serves to minimize the risk of failure by enabling early problem diagnosis on a smaller scale, allowing us to refine our strategy for the subsequent steps. Additionally, it helps to limit material and labor loss in case of any unforeseen challenges. All 5-L–500-L bioreactors were operated in fed-batch mode.

### Cell culture

The recombinant CHO K1 cells expressing the antibody-based therapeutic (an anti-TNFα IgG1 antibody) were obtained from Shanghai Zhanjiang Biotechnology Co., Ltd, Shanghai, China. The cell line culture process comprised cell recovery, cell expansion, and a final fed-batch culture. The culture was grown in a serum free medium (CHOM-B03) and equilibrated with a 5% CO_2_/air mixture overnight before seeding. It was maintained in a CO_2_ incubator with a 5% CO_2_/air mixture at 37°C. For the 5 L scale culture, primary seeds were shake flask amplified and after expansion to 0.9 L, seeds were transferred to a 5 L bioreactor at a 1:4.4 inoculation ratio to initiate the culture. For the 50 L scale culture, primary seeds were first expanded in shake flasks, followed by a secondary expansion in 5 L bioreactors. Once the culture volume reached 5 L, it was transferred to a 50 L bioreactor at a 1:4 inoculation ratio. The culture was maintained until day 2, at which point a second feed expanded the volume to 40 L, continuing thereafter with fed-batch cultivation. For the 500 L scale culture, the seeding process mirrored that of the 50 L scale for the initial two stages, with the third stage of seed expansion occurring in a 50 L bioreactor. After the culture volume increased to 50 L, it was transferred to a 500 L bioreactor, also at a 1:4 inoculation ratio. On the second day, the culture was fed twice, reaching a total volume of 400 L, and was then maintained using the fed-batch cultivation method. The batch inoculation density should be approximately 1.0-1.2×10^6^ cells/mL, and the cell viability should not be less than 90%. Cell density, viability, cell status, and glucose levels were measured daily during the culture period. Product expression was measured on days 8, 10, 12, 14, and 16, and product quality attributes were measured after purification by affinity chromatography.

### Bioreactor control

The incubation period lasted for 16 days during which the temperature conditions were set at 37°C for day 0 to day 7 and 34°C for day 8 to day 16. The pH was meticulously regulated within the range of 6.7–7.2 using a combination of CO_2_ and sodium carbonate. Dissolved oxygen (DO) levels were consistently kept at 40% air saturation across all IFSBs by means of interaction between the gas-exposed smooth surface of the vessel and the culture medium inside. In he STRs, the DO level was controlled by introducing air at a rate of 1.7 mL/min and oxygen at a rate of 1–10 mL/min. This rate was automatically adjusted based on the DO level. For 5-L, 50-L and 500-L IFSBs, the rocking speeds were consistently maintained at 50–52 rpm, 32–33 rpm and 21.5–24.5 rpm respectively. Simultaneously, the stirring speed of the 5-L STR was held within the range of 230–260 rpm.

### Analytical assays

#### Measurement of nutrient concentrations, titer, cell count, and related calculations

We measured glucose levels with a glucose assay kit using the glucose oxidase method. The monoclonal antibody titer was determined through Protein A-affinity HPLC. Cell density, viability, and diameter were assessed with a ViCell cell viability analyzer. Viable and non-viable cell counts were obtained using cells prestained with trypan blue. The integral viable cell density (IVCC), calculated by summing areas under each cell count using the trapezoid rule, represents the integral area under the VCD *versus* time curve. Specific cell productivity (pg/cell/day) is determined by dividing the total antibody amount in the bioreactor by the IVCC.

#### High-performance liquid chromatography (HPLC) analysis

Charge variants were analyzed using Ion Exchange High-Performance Liquid Chromatography (IEX-HPLC), while aggregates were assessed through Size Exclusion Chromatography with Ultra-Performance Liquid Chromatography (SEC-UPLC). The standard antibody-based biotherapy, stored at −80°C, was frozen and subsequently diluted to a concentration of 5.0 mg/mL using the mobile phase. The resulting solution underwent centrifugation at 5,180 *g* for 10 min, and 20 μL of the supernatant was subjected to analysis. For a sample concentration within the range of 1–5.0 mg/mL, a dilution with the mobile phase was performed, followed by centrifugation at 5,180 *g* for 10 min. Post-centrifugation, the supernatant was collected and analyzed. The column temperature was maintained at 25°C, the acquisition wavelength was set to 280 nm, with a sampling frequency of 5 times/second. The sample chamber temperature was controlled at 10°C ± 5°C.

### Antibody purification

The purification process followed standard antibody purification protocols, which involved protein A capture followed by polishing steps. Initially, proteins were purified using affinity chromatography with an AutoPrep column system, which was equilibrated with a solution containing 20 mM Tris, 150 mM NaCl, and a pH range of 6.8–7.2. The flow rate during this step was maintained at 1.0 mL/min. The culture supernatant was then filtered through a 0.22 μM filter membrane, up-sampled, and subsequently eluted using an elution solution consisting of 25 mM Citrate and a pH range of 2.8–3.2. The equilibrium and eluent used for cation exchange chromatography consisted of a 20 mM solution of sodium acetate-acetic acid, 30 mM of NaCl, with a pH range of 4.8–5.2 by 1.2 mL/min. Similarly, for hydrophobic chromatography, the equilibrium and eluent comprised a 20 mM solution of sodium acetate-acetic acid, 300 mM of NaCl, with a pH range of 4.8–5.2 by 1.5 mL/min.

### Scale-up methodology

#### Operational parameters scale-up

The operational parameters, including temperature, DO, and pH, were found to be independent of volume. Therefore, these parameters for large vessels were configured to match the values established for the 5-L IFSB. Maintaining pCO_2_ levels within the range of 15–180 mmHg at all scales was crucial, as cell growth outside this range was inhibited in the 5-L scale. Scaling up the total air flow rate was a straightforward process, relying on the normalized volumetric flow rate in relation to the vessel working volume. This involved dividing the total air flow rate by the vessel working volume (Eq. 1) (Redl et al., 2017). The flow rates of carbon dioxide, oxygen, and superficial airflow were adjusted based on the levels of dissolved oxygen and pH. It was essential to set the line pressures for carbon dioxide and oxygen slightly higher than the air line pressure to ensure proper injection of both gases into the air stream. Achieving this required setting the gas line pressure using a pressure regulator.

$$V_{s}=Q_{\text{gas}}/A_{v}$$ (1)

V_s_: Superficial gas velocity,

Q_gas_: Volumetric gas flow rate,

A_v_: Area of the bioreactor.

Scaling up agitation speed, on the other hand, is relatively simpler compared to traditional STRs. The speed is determined based on a constant Froude Number (Eq. 2). The Froude number, a dimensionless quantity representing the ratio of inertial force to gravity, is crucial. The IFSB is calculated as the ratio of centrifugal force to gravity, playing a key role in driving IFSB movement. It significantly influences flow characteristics, mixing effects, oxygen transfer, and shear force in the bioreactor. The new speed must ensure sufficient mixing in large vessels without causing excessive agitation or aeration-related damage to the culture (Thompson et al., 2019).

$$Fr=\frac{2\cdot \pi \cdot n\cdot \left(\frac{d_{i}+d_{0}}{2}\right)}{\sqrt{d_{i}\cdot g}}$$ (2)

*d*_*i*_ is the maximum diameter of the IFSB (mm), *d*_*0*_ is the amplitude diameter of the IFSB (mm), n is the speed of the IFSB (rpm), *g* is the gravitational acceleration (m/s^2^).

#### Feeding parameters scale-up

The culture process was fed from day 2. The feed media CHOM-S03 and CHOM-S04 were added in proportions of 1.5% and 0.15%, respectively, of the initial volume at 37°C. When the glucose concentration fell below 4 g/L, it was supplemented to 5 g/L daily. The schedule for feed additions remained consistent irrespective of the bioreactor volume, mirroring that of the 5-L counterpart. However, adjustments to the feed volume and feed rate should be made based on a scale factor that accounts for volume differences.

#### Sampling scale-up

Sampling frequency and sample size were determined for each scale. With each sample, there was a loss in volume from the bioreactor. While this may not pose an issue for larger vessels, it could lead to problems in small-scale bioreactors as each sample represents a more substantial percentage of the total bioreactor volume. Such losses need careful consideration when formulating a scale-up strategy. In this study, samples were taken at least once per day for all scales, with a volume of approximately 40 mL for the 5-L scale and 200 mL for all larger scales.

#### Inoculation scale-up

The post-inoculation cell density in the bioreactor remained volume-independent; thus, it was consistently maintained across scales. Additionally, the inoculation percentage (v/v) was closely regulated to ensure a comparable volume increase after inoculation. The *in vitro* age of the inoculum, from the date of thawing from the Cell Bank to the bioreactor inoculation date, was also controlled within a similar range across scales, ensuring that the cells initiated the process at a comparable biological status. In this study, the inoculation ratio ranged between 1:4 and 1:5. The *in vitro* age for all large-scale studies was controlled within a 1-month timeframe.

### Experimental design

Initially, the process performance parameters and product quality attributes were compared between the IFSB and STR at a 5-L scale, providing essential technical reference data for scaling up cultivation. Operational parameters were then determined for the 50-L and 500-L IFSBs based on the proposed scaled-up principles, and the transition to larger bioreactors was conducted in a stepwise manner. Bioprocess performance and product quality were compared across scales to further assess the outcomes of the scale-up process.

### Shear force simulation calculation

To investigate the flow field and assess the performance of the IFSB, computational fluid dynamics (CFD) simulations were employed to analyze the shear force acting on the IFSB. The detailed simulation methodology is outlined in the Supplementary Material. The IFSB is propelled by an oscillator, which can be conceptualized as a rigid body undergoing horizontal rotation with eccentricity on an oscillating platform. The trajectory of each point on the IFSB remains consistent, ensuring uniform speed and acceleration.

## Results

### Process parameters

**FIGURE 1:**
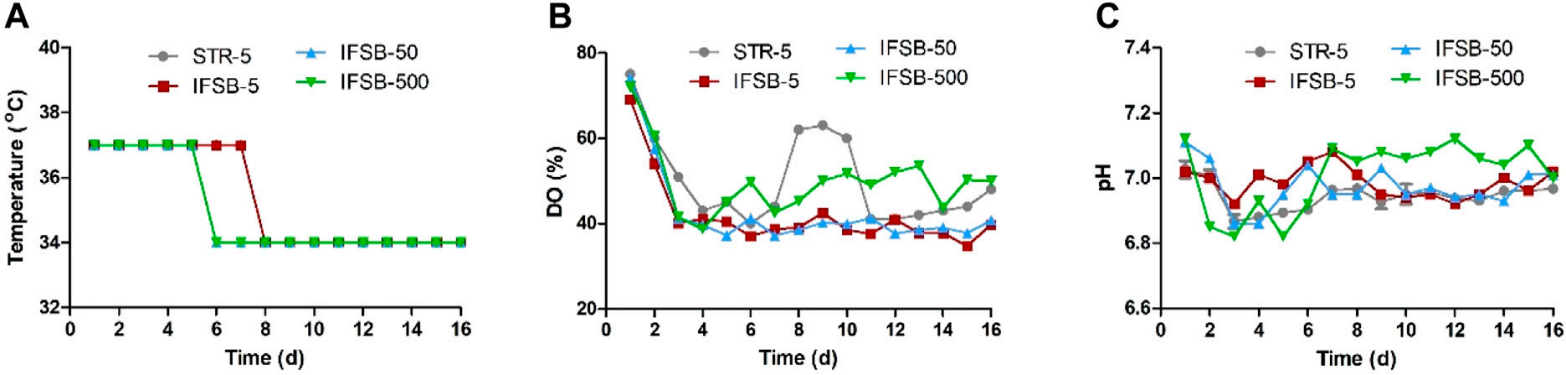
Typical control profiles of vessel temperature, DO and pH in different large-scale bioreactor. **(A)** Temperature controlled in different large-scale bioreactor, **(B)** DO controlled in different large-scale bioreactor, **(C)** pH controlled in different large-scale bioreactor.

The scale-independent process parameters, such as medium, temperature, pH, DO, feed regimen, seeding density, and culture cycle, remained consistent across different scales. Figures 1A–C illustrates the typical control profiles of the bioreactor temperature, pH, and DO for STR-5, IFSB-5, IFSB-50, and IFSB-500. The temperature profiles were effectively maintained around the set point. For STR-5 and IFSB-5, it was 37°C from day 1 to day 7°C and 34°C from day 8 to day 16. In the case of IFSB-50 and IFSB-500, the temperature was 37°C from day 1 to day 5°C and 34°C from day 6 to day 16. The DO profiles exhibited a declining phase followed by a control phase, marked by band-like spikes around the set point of 40% air saturation due to oxygen supplementation. pH profiles fluctuated within the set range of 6.9–7.2 for all scales.

The scale-dependent process parameters, including agitation speed, batch volume, and gas flow rate, are presented in Table 1. The scale-dependent process parameters for the 5-L STR refer to the previously well-established and stable cultivation process. The stirring speed and surface air flow rate for IFSB-5 were set at 50 rpm and 1.7 mL/min, respectively, based on the characteristics and application experience of IFSBs. The O_2_ flow rate and CO_2_ flow rates were determined according to the DO and pH levels of the culture medium, ranging from 0 to 10 mL/min, respectively. The scale-dependent process parameters for IFSB-50 and IFSB-500 are also listed in Table 1. The suggested agitation speeds were calculated based on the 50 rpm agitation speed set in the 5-L bioreactor, using Eq. 2. Additionally, the O_2_ flow rate and CO_2_ flow rates were calculated based on a 5-L bioreactor, using Eq. 1.

**TABLE 1:** The scale-dependent process parameters for the processes.

Criteria	STR-5	IFSB-5	IFSB-50	IFSB-500
Agitation speed (rpm)	230–260	50–52	32–33	21.5–24.5
Deep air flow rate (L/min)	1.7	N/A	N/A	N/A
O_2_ flow rate (L/min)	0–10	0–0.1	0–0.5	0–10
CO_2_ flow rate (L/min)	0–10	0–0.1	0–0.5	0–5
Superficial air rate (L/min)	0.05–0.5	0.1	0.5	1.5
Batch volume L)	4	4	40	400

### Comparison of the bioprocess performance

**FIGURE 2:**
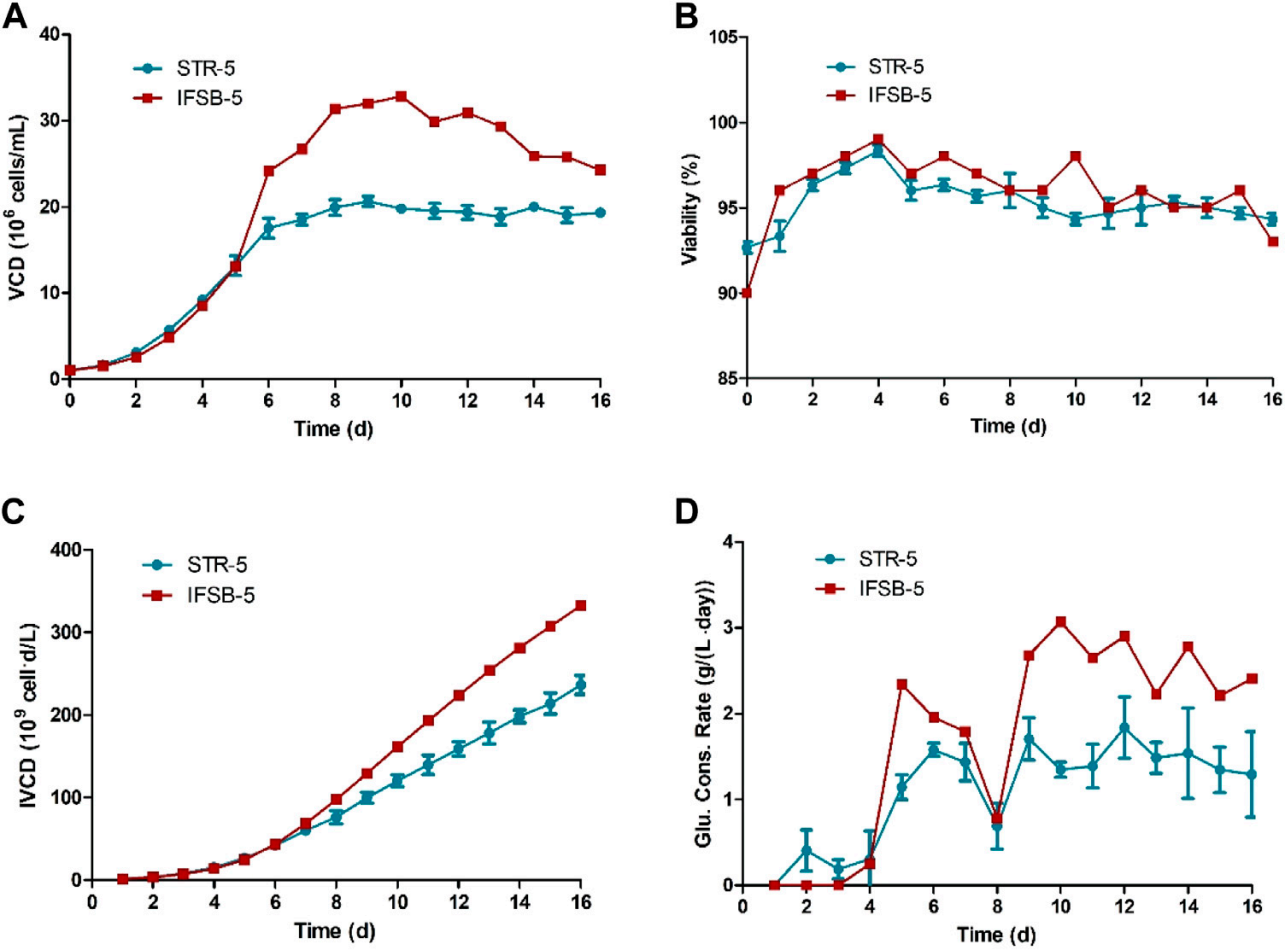
Comparison of the culture growth profiles of STR-5 and IFSB-5. **(A)** VCD; **(B)** Viability; **(C)** IVCD; **(D)** Glucose consumption profile. Values are means ± SD, *n* = 3 for STR-5, one experiment for IFSB-5.

The growth, product formation, and performance of STR-5 and IFSB-5 cultures were assessed by comparing their growth profiles, glucose consumption, and antibody production. IFSB-5 exhibited a superior maximal VCD compared to STR-5 (3.3 × 10^7^ vs 2.2 × 10^7^ viable cells/mL, on average). Cell viability remained consistently high in both bioreactors, surpassing 90%, with no significant differences observed. The integral of the viable cell concentration over time (IVCD) for IFSB-5 progressively exceeded that of STR-5. To maintain optimal conditions, glucose concentration was sustained and supplemented at 5 g/L whenever it fell below 4 g/L. Illustrated in Figure 2, IFSB-5 demonstrated a higher daily glucose consumption than STR-5 from day 9 onwards, attributed to its elevated VCD.

**FIGURE 3:**
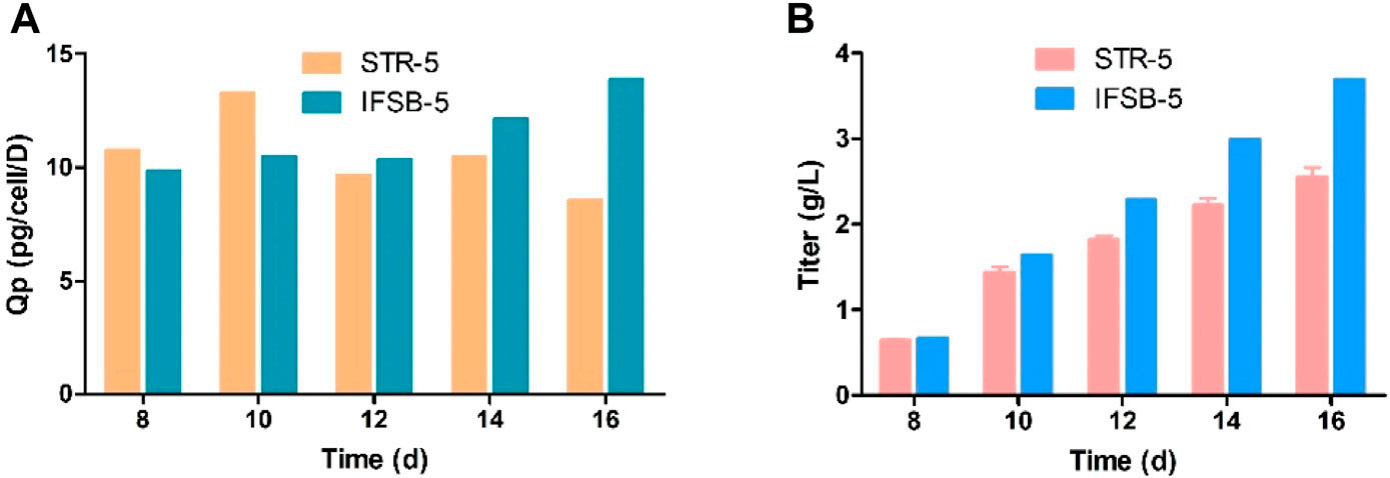
Comparison of the antibody production of STR5 and IFSB-5. **(A)** Specific productivity (Qp) after day 8. Data shown are the average of three independent experiments for STR-5, an experiment for IFSB-5, **(B)** Titer after day 8, Data shown are the average of three independent experiments for STR-5 (mean ± SD), one experiment for IFSB-5.

In Figure 3, antibody production was measured from day 8 onwards in the culture. The results indicated a gradual increase in antibody concentration, with IFSB-5 showing a higher final antibody concentration (3.70 g/L) compared to STR-5 (2.56 g/L). The specific productivity (Qp) remained comparable for both IFSB-5 and STR-5, suggesting that the enhanced antibody production was attributed to an increase in the cumulative cell count.

### Comparison of product quality attributes

The quality of the various products was assessed by examining critical attributes of antibody-based biotherapeutics, including aggregation, structural integrity, and charge variants.

Antibody aggregates, formed through intermolecular forces during production, pose potential harm, triggering immune responses in humans, leading to hypersensitivity reactions (Lundahl et al., 2021). Such aggregates, if present during intravenous administration, may even lead to capillary blockage and reduced microcirculation. Therefore, controlling aggregates is crucial for the development and production of protein-based drugs. The HPLC-SEC results indicated that the IFSB-5 product had a purity of 99.4% with 0.6% aggregates, while the STR-5 product had a purity of 99.3% with 0.7% aggregates (Table 2). The presence of aggregates in both bioreactors was minimal.

**TABLE 2:** The product quality attributes of STR-5 and IFSB-5.

Bioreactor	SEC (%)		IEX (%)			High mannose (%)
	Aggregates	Main peak	Acidic variants	Main peak	Basic variants	
STR-5	0.7 ± 0.15	99.3 ± 0.17	27.0 ± 0.89	54.6 ± 0.25	18.4 ± 0.65	2.10 ± 0.10
IFSB-5	0.6	99.4	21.5	59.1	19.4	2.14

SEC: size exclusion chromatography; IEX: Ion exchange chromatography. Data shown are the average of three independent experiments for STR-5 (mean ± SD), one experiment for IFSB-5.

Charge variants, categorized as acidic and basic, can impact various aspects of antibody-based biotherapeutics. These include binding activity, bioactivity, pharmacokinetics, and structural stability (Beck et al., 2022). The effect of bioreactors on product quality was further assessed by analyzing charge variants using IEX HPLC. IEX HPLC results showed that the basic variants of IFSB-5 and STR-5 were comparable (19.4% vs 18.4%, respectively), while the acidic variants of IFSB-5 showed improvement (21.5% vs 27.0%, respectively). The main peak of IFSB-5 was higher than that of STR-5 (59.1% vs 54.6%) (Table 2).

Fc glycosylation profiles significantly impact the clinical efficacy and safety of antibody-based biotherapeutics. Mannose-type N-glycans may affect efficacy, pharmacokinetics, immunogenicity, and stability. Marketed antibody-based biotherapeutics exhibit significantly higher levels of mannose glycosylation compared to endogenous biotherapeutics (Mastrangeli et al., 2020). Examination of high mannose glycosylation levels revealed that IFSB-5 and STR-5 had similar levels of high mannose-type N-glycans (2.14% vs 2.10%, respectively), both displaying low levels of high mannose glycosylation (Table 2).

### Bioprocess performance of IFSB at different scales

**FIGURE 4:**
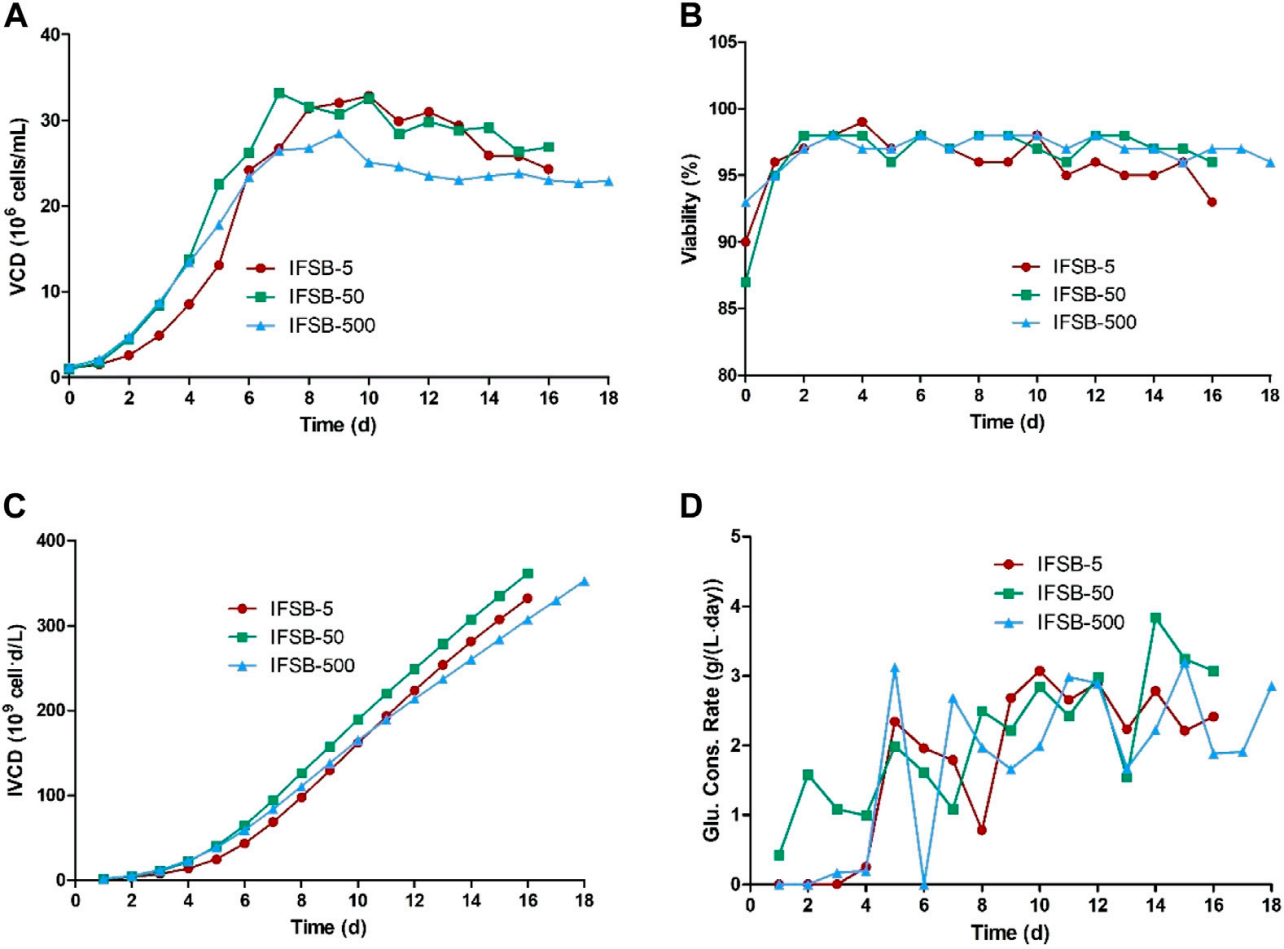
Comparison of the culture growth profiles of IFSB-5, IFSB-50 and IFSB-500. **(A)** VCD, **(B)** Viability, **(C)** IVCD, **(D)** Glucose consumption profile. One experiment in each scale.

The bioprocess performance and product quality of the IFSB at a 5-L scale were comparable to or even better than those of a STR. Therefore, the scalability of the IFSB process was further assessed at 50-L and 500-L scales. The growth and product formation at each scale were qualitatively evaluated by comparing the profiles of growth, glucose consumption, and antibody production. The results indicated that the maximum VCD of IFSB was similar at all scales, with average values of approximately 3.29 × 10^7^, 3.32 × 10^7^, and 2.85 × 10^7^ viable cells/mL for IFSB-5, IFSB-50, and IFSB-500 respectively (IFSB-500 exhibited a slightly lower VCD). There was no significant change in cell viability or IVCD between the scales, with cell viability consistently remaining above 90%. Glucose concentration was effectively controlled though daily supplementation when it fell below 4 g/L. The culture cycle for IFSB-500 was extended to 18 days due to its excellent cell status on day 16 (Figure 4).

**FIGURE 5:**
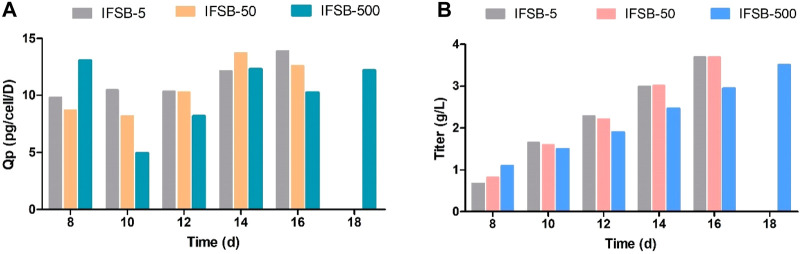
Comparison of the antibody production of IFSB-5, IFSB-50 and IFSB-500. **(A)** Specific productivity (Qp) after day 8; **(B)** Titer after day 8. One experiment in each scale.

As illustrated in Figure 5, the assessment of antibody production revealed a consistent final product titer between IFSB-5 and IFSB-50 (3.70 g/L for both). However, IFSB-500 exhibited a slightly lower titer on day 16 (2.95 g/L vs 3.70 g/L), possibly attributed to the lower VCD. Nevertheless, due to the robust cell condition, the culture cycle was extended to 18 days, resulting in IFSB-500 achieving a final titer of 3.51 g/L comparable to IFSB-5 on day 16.

### Product quality of IFSB at different scales

The comparability of the scaled-up process with the IFSB process was further assessed through analytical testing of the products generated at each scale. The results indicated that the purities of IFSB-5, IFSB-50, and IFSB-500 were comparable, exhibiting similar acidic variants (21.5%, 20.6%, and 22.7%), basic variants (19.4%, 18.8%, and 22.4%), and high mannose-type N-glycans (2.14%, 2.26%, and 1.85%, respectively). Minimal aggregation levels were observed, and these decreased further at the IFSB-500 scale. Extending the culture period to 18 days resulted in an increase in acidic variants (from 22.7% to 25.3%), while basic variants remained stable (Table 3).

**TABLE 3:** The product critical quality attributes of IFSB-5, IFSB-50 and IFSB-500.

Bioreactor	SEC (%)		IEX (%)			High mannose (%)
	Aggregates	Main peak	Acidic variants	Main peak	Basic variants	
IFSB-5	0.6	99.4	21.5	59.1	19.4	2.14
IFSB-50	1.1	98.9	20.6	60.6	18.8	2.26
IFSB-500 (D16)	0.3	99.7	22.7	54.9	22.4	1.85
IFSB-500 (D17)	0.8	99.2	23.9	53.4	22.7	1.88
IFSB-500 (D18)	0.8	99.2	25.3	52.2	22.5	2.19

SEC, size exclusion chromatography; IEX, Ion exchange chromatography. One experiment in each scale.

### Shear force simulation calculation

Given an IFSB with good process performance and scalability, this study simulated the shear of an IFSB using CFD fluid simulation. The Supplementary Material provides a detailed description of the simulation methodology. The results indicate that the volume-averaged shear rate of the IFSB tends to decrease with increasing reactor volume. Furthermore, the volume-averaged shear rate for all IFSB sizes was found to be less than 20 s^-1^. The shear rate generated by the IFSB is significantly lower than the maximum shear rate that mammalian cells can withstand. As a result, the damage caused by IFSBs to CHO cells can be neglected (Born et al., 1992; Chalmers and Ma, 2015). Therefore, IFSBs meet the mechanical requirements of mammalian cell cultures.

## Discussion

The development of high-density cell culture systems has enhanced the cost-effectiveness of monoclonal antibody production, establishing antibody-based biotherapeutics as prominent therapeutic and diagnostic proteins in the biopharmaceutical industry (Jyothilekshmi and Jayaprakash, 2021). To further enhance protein quality and production output for commercial purposes, new bioreactor types are necessary. This study assessed the product quality and productivity of an antibody-based biotherapeutic culture in IFSB and STR bioreactors, showcasing high cell viability (>90%) under specific conditions. However, the IFSB exhibited a higher VCD during the plateau phase, resulting in a 51.1% increase compared to the STR. Consequently, the IVCD of IFSB-5 was progressively higher than that of STR-5, and the IFSB-5 bioreactor consumed more glucose daily. While the specific unit productivities of STR and IFSB at the 5-L scale in the pre-culture period were similar, the specific productivity of STR showed a slight decrease, whereas that of IFSB exhibited an increasing trend. IFSB demonstrated 62.4% higher productivity than STR, indicating that the cells in IFSB maintained a good status throughout the culture. Eventually, protein expression in the IFSB reached 3.70 g/L, reflecting a 44.5% increase compared to that in the STR. Increased molecular complexity can lead to higher levels of protein aggregates, charge variants, and physical and chemical degradation in cell culture (Gomez et al., 2020). Further evaluation is required for quality attributes, such as aggregates and charge variants. The evaluation results showed that the proteins in IFSB exhibited properties similar to those of STR in terms of aggregates and basic variants, with improved acidic variants.

The protein expression in IFSB exhibited increased levels, and the product quality either remained comparable or showed improvement. This promising outcome prompted a more in-depth assessment of the IFSB scale-up process. The challenge of scaling up biological processes lie in the intricate interplay of various, sometimes conflicting, factors. Efficient mammalian cell culture hinges on two crucial elements: mixing and oxygen transfer. However, the operation of systems designed to achieve this efficiency is complex. High-cell-density culture systems, while challenging to operate, can yield compact bioreactors with elevated production levels (Ozturk, 1996). However, IFSBs prove to be relatively simple in both scalability and operation. The scale-up of agitation speed in IFSBs was validated using the principle of equality of the Froude number. This principle played a pivotal role in propelling the movement of the IFSB, significantly impacting flow characteristics, mixing effects, oxygen transfer, and shear force within the bioreactor (Blunt et al., 2019). Scaling up the total airflow rate in IFSBs was straightforward, as it was based on the working volume. Carbon dioxide and oxygen flow rates were initially set at the same rate as the total airflow, although adjustments may be necessary depending on the type of DO controller in the bioreactor and the chosen control strategy.

The results from the scaled-up culture of IFSB reveal that the process performance of the IFSB-5 and IFSB-50 bioreactors was comparable, achieving a protein expression of 3.6 g/L, with consistent protein quantity. This implies that the scaled-up process of the IFSB-50 bioreactor remained stable. The IFSB-500 bioreactor exhibited initial cell growth similar to that of IFSB-50, but with a lower plateau VCD. The final protein expression in the IFSB-500 bioreactor was 2.9 g/L, representing 83% of that in IFSB-50. However, both VCD and protein expression levels surpassed those observed in STR-5. The extended culture period of IFSB-500 to 18 days, driven by its high VCD and cell viability on day 16, resulted in an increase in protein expression to 3.5 g/L after a 2-day extension, aligning with the levels observed in IFSB-50. Addressing large-scale global optimization proves challenging due to factors such as high dimensionality, an extensive search space, an unknown landscape, irregularity, and the existence of numerous local optimal solutions (Liu et al., 2023). Nevertheless, utilizing a scaled-down model for subsequent optimization can enhance the consistency of scaled-up production in the future. These findings suggest that IFSB minimizes shear force on cells, maintains superior VCD and cell viability, and facilitates higher product expression over an extended culture period.

IFSB-50 and IFSB-5 exhibited consistent protein quality. Culturing with IFSB-500 extended the culture period by 2 days. The proportion of acidic variants in the product gradually increased, while the levels of basic variants and high mannose content remained stable. This underscores the advantages of using IFSBs as antibody-based biopharmaceuticals to enhance protein productivity and quality. However, challenges arise when scaling up IFSBs beyond 2000 L. Nevertheless, it has successfully expanded its production flexibility.

## Conclusion

The use of IFSBs in cell culture holds the potential to minimize shear damage to cells, enhance cell density, and improve cell status. This, in turn, can result in increased productivity and product quality comparable to or better than traditional STRs. Employing a stepwise approach for scaling up, progressing from a 5 L culture to 50 L and 500 L cultures, enhances the likelihood of success. The scaled-up IFSB demonstrated stable operation and consistent process parameters. Product expression remained at approximately 3.0 g/L, and the quality attributes of the product remained consistent. These findings suggest that IFSBs are viable bioreactors with favorable scalability for antibody-based biotherapeutics in CHO cells.

## Data Availability

The original contributions presented in the study are included in the article/Supplementary Material, further inquiries can be directed to the corresponding authors.
