# Supplementary material for: Improving product quality and productivity of an antibody-based biotherapeutic using inverted frustoconical shaking bioreactors
Source: Front Bioeng Biotechnol. 2024 Mar 22;12:1352098. doi: 10.3389/fbioe.2024.1352098 (PMC10995296; doi:10.3389/fbioe.2024.1352098)
Supplement: Supplementary file 1 [file DataSheet1.DOCX]

**Supporting Information**

**Improving Product Quality and Productivity of an Antibody-based Biotherapeutic Using Inverted Frustoconical Shaking Bioreactors**

Xuekun Wang^1,2,†^, Jin Xu^1,2,3,†^, Qingcheng Guo^1,2,3,4,†^, Zhenhua Li^1,2,5^, Jiawei Cao^1,2,5^, Rongrong Fu^1,2^, Mengjiao Xu^1,2^, Xiang Zhao^1,2^, Fugui Wang^1,2^, Xinmeng Zhang^1^, Taimin Dong^1^, Xu Li^1^, Weizhu Qian^1,2,3^, Shen Hou^1,2,3^, Lusha Ji^1,2,3,*^, Dapeng Zhang^1,2,3,*^, Huaizu Guo^1,2,3,4,*^

^1^State key laboratory of macromolecular drugs and large-scale manufacturing, School of Pharmaceutical Sciences, Liaocheng University, Liaocheng, China

^2^NMPA Key Laboratory for Quality Control of Therapeutic Monoclonal Antibodies, Shanghai, China

^3^State key laboratory of macromolecular drugs and large-scale manufacturing, School of Pharmaceutical Sciences, Wenzhou Medical University, Wenzhou, China.

^4^Taizhou Mabtech Pharmaceuticals Co., Ltd, Taizhou, China

^5^State key laboratory of macromolecular drugs and large-scale manufacturing, Shanghai Zhangjiang Biotechnology Co., Ltd, Shanghai, China

^†^These authors contributed equally to this work.

^*^Correspondence to: Lusha Ji, Dapeng Zhang and Huaizu Guo

E-mail: jilusha@lcu.edu.cn, bigbirdzcn@163.com and guohuaizu@163.com

The simulation was conducted by a computational fluid dynamics (CFD) software FLUENT, and the simulated process was as follows.

**1. Construction of IFSB**

The flow field in the inverted frustoconical shaking bioreactor (IFSB) is mainly related to the structural dimensions of the reactor tank, and the geometry of the bioreactor is shown in Figure S1. The bioreactor is mainly composed of the upper part of the cylinder and the lower part of the vertebrae, with the diameter of the cylinder D1, the diameter of the vertebrae D2, and the vertebrae half-cone apex angle α.


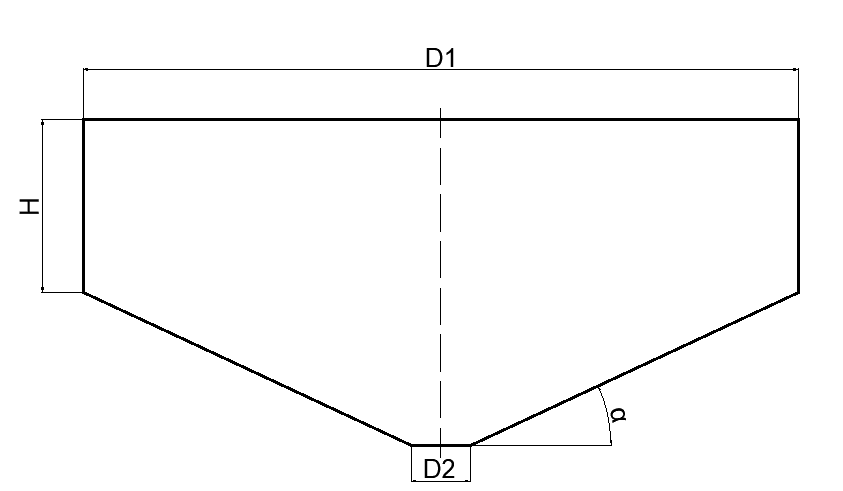


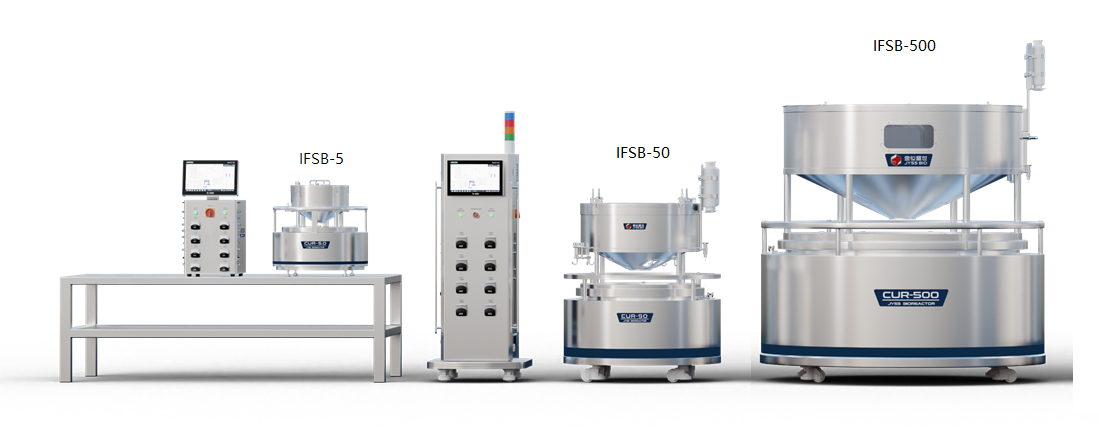


Figure S1 The schematic diagrams of bioreactors

Three different sizes of IFSB (IFSB-5, IFSB-50, and IFSB-500) were computationally simulated and the geometric parameters of IFSB are shown in Table S1. The IFSBs is driven by an oscillator, which can be abstracted as a rigid body making horizontal eccentric rotations on an oscillating platform, with the same trajectory at each point, and the same velocity and acceleration. Table S1 shown the eccentricity of the eccentric rotation of the IFSBs.

Table S1 Geometric parameters of IFSB tanks

| Scale | D1  （mm） | D2  （mm） | H1  （mm） | Eccentricity  （mm） | Half-cone  angle（^o^） | Speed  （rpm） |
| --- | --- | --- | --- | --- | --- | --- |
| IFSB-5 | 410 | 79.14 | 122 | 30 | 32 | 55 |
| IFSB-50 | 850 | 160 | 260 | 40 | 26.5 | 40 |
| IFSB-500 | 1700 | 190 | 420 | 65 | 25 | 28 |

**2 CFD modeling of** IFSB

*2.1* *Determination of fluid properties in IFSB*

The medium used for mammalian cell culture is mainly composed of water, so the rheological properties of medium and water are close to each other, and water is used instead of medium in the simulation model. Oxygen passed into the bioreactor is replaced by air, and the fluid properties of water and air are shown in Table S2.

Table S2 Properties of water and air in IFSB

| Substance | Temperature  (^o^C) | density  (Kg/m^3^) | viscosity（Kg/m*s） | Surface tension coefficient（N/m） |
| --- | --- | --- | --- | --- |
| H_2_O | 20 | 998.2 | 0.001003 | 0.0728 |
| air | 20 | 1.225 | 1.7894e-5 |  |

*2.2* *Delineation of the computational grid*

In this study, the Mesh function in Workbench was applied to mesh the IFSB, as shown in Figure S2, and a tetrahedral unstructured mesh was utilized, which was certified for mesh-independence, and the final size and sparsity of the division were determined, as shown in Table S3.


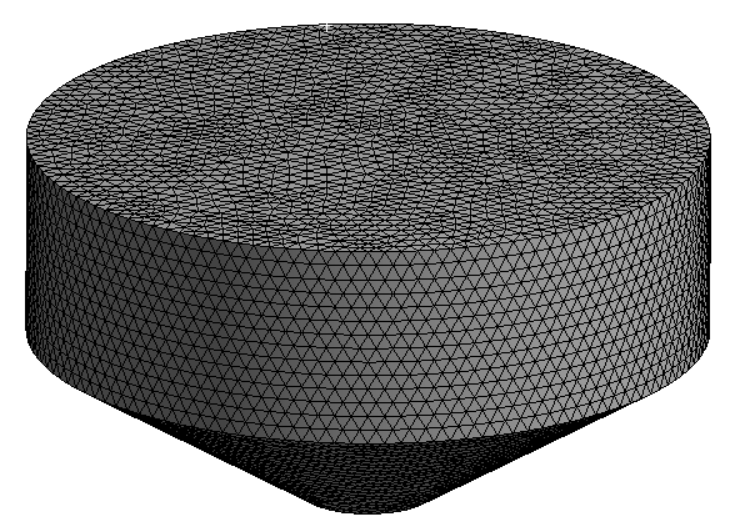


Figure S2 Schematic mesh delineation of IFSB

Table S3 Grid information of IFSB-

| Scale | Maximum size (mm) | Node  (Node) | Grid  (Element) | minimum mesh quality  (Min) | average mesh quality  (mean) |
| --- | --- | --- | --- | --- | --- |
| IFSB-5 | 5 | 90486 | 479838 | 0.44 | 0.84 |
| IFSB-50 | 10 | 95772 | 506801 | 0.41 | 0.84 |
| IFSB-500 | 20 | 86226 | 455183 | 0.42 | 0.84 |

*2.2* *Mathematical Models and Calculation Methods*

The IFSB involves two-phase flow of air and water, so the VOF model is used. The advantage of the VOF model is mainly the tracking of the air-liquid free surface, which can well represent the fluid motion state at the air-liquid interface. As shown in Figure S3, area A is air, area C is water, and B is the air-liquid interface area. The RNG k-ℇ model is used for the turbulence model.


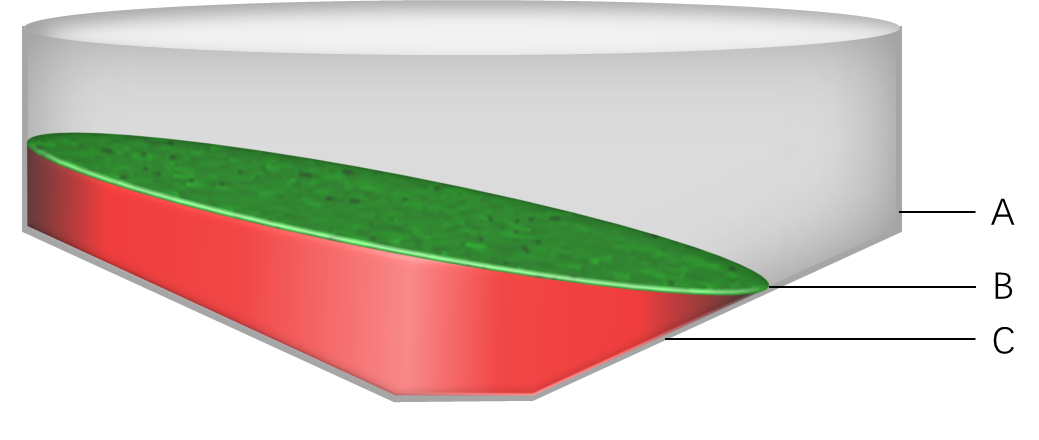


A

B

C

Figure S3 Schematic diagram of VOF model for IFSB

For the motion boundary of the IFSB, the motion of the culture medium comes from the bioreactor doing eccentric motion in the plane, so the whole reactor tank is used as the computational domain. In this report, dynamic grid and UDF are used to define the boundary motion of the IFSB, and the equations of motion are shown below:

$$\begin{matrix} V_{\text{x}}=R\omega\cos(\omega t+\phi_{0}) \\ V_{\text{y}}=-R\omega\sin(\omega t+\phi_{0}) \end{matrix}$$

$V_{\text{x}}$: The linear velocity of the point in the x-direction (m/s);

$V_{\text{y}}$: The linear velocity of the point in the y-direction (m/s);

R: Eccentricity of IFSB;

$\omega$: Angular velocity of IFSB (rad/s);

$\phi_{0}$: Initial phase angle of the point (rad).

The model is computed transiently with the time step set to 0.005s and the iterative residuals set to 0.001.

**3 Results of CFD**

The average shear rate within the flow field is analyzed through fluid simulation. The average shear rate is a volume integral of the shear rate over the entire region of the flow field and then averaged. It is calculated by the formula:

$$\bar{\tau}=\frac{1}{\sum_{i-1}^{\text{n}} V_{i}}\sum_{i-1}^{\text{n}} V_{i}\cdot V_{i}$$

$\overline{\tau}$: The average shear rate;

n: Total number of grids;

$\tau_{\text{i}}$: Shear rate of the i'th grid;

$\text{V}_{\text{i}}$: Volume of the i'th grid.

The results of the average shear rate of the IFSB for different working volumes and different rotational speeds are shown in Figure S4. The results show that in IFSB, the average shear rate tends to increase with increasing rotational speed for a constant working medium, and decreases with increasing working volume at the same rotational speed. In the three types of IFSBs, IFSB-5, IFSB-50, and IFSB-500, the average shear rate tends to decrease with increasing reactor volume, indicating that the effect on the cellular damage also decreases.


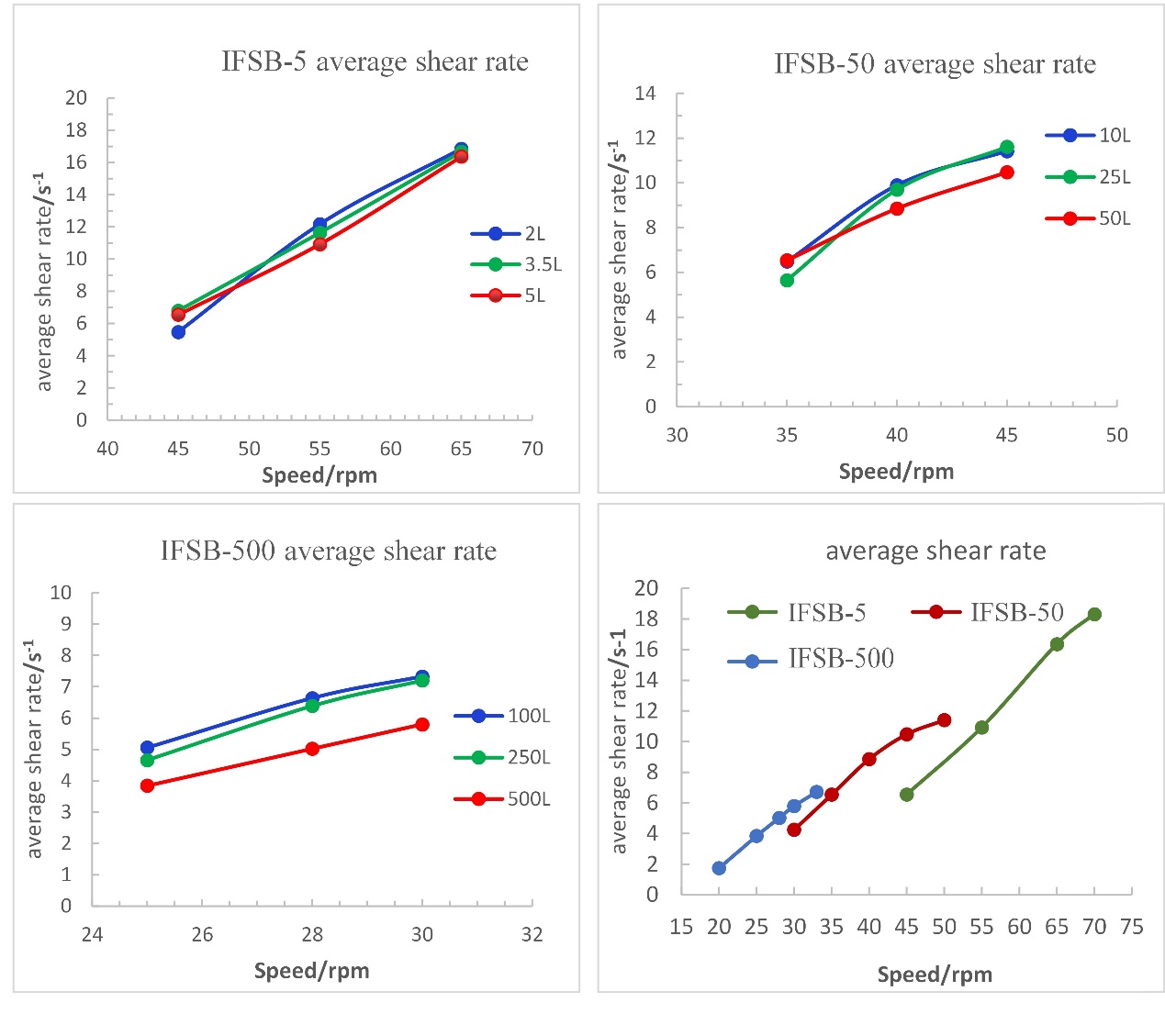


Figure S4 Average shear rate for CFD simulation of IFSB
